# Measuring mental health action competencies in school teachers: internal and external validity evidence

**DOI:** 10.3389/fdgth.2024.1257392

**Published:** 2024-02-13

**Authors:** Matthew J. Kerry, Dominik Robin, Kurt Albermann, Julia Dratva

**Affiliations:** ^1^ Institute of Public Health, ZHAW Zurich University of Applied Sciences, Winterthur, Switzerland; ^2^ Centre of Social Paediatrics, Cantonal Hospital Winterthur, Winterthur, Switzerland; ^3^ Medical Faculty, University of Basel, Basel, Switzerland

**Keywords:** mental health, health literacy, item response theory (IRT), bifactor, adolescent health, child health

## Abstract

**Introduction:**

Mental health literacy is receiving increasing research attention due to growing concerns for mental health globally. Among children, teachers have recently been recognized as playing a vital role in the recognition and reporting of potential mental health issues.

**Methods:**

A nationally sampled cross-section of teachers was surveyed to examine the discriminant validity of the mental health literacy measure across levels of teaching. A survey collected a total of *n = *369 teacher responses in Switzerland (Kindergarten = 76, Primary = 210, Secondary = 83). Item response theory (IRT) analyses were conducted.

**Results:**

Inspection of psychometric properties indicated removal of two weak performing items. The 15-item measure exhibited a significant mean difference, such that class-responsibility function scored higher (*M *= 2.86, *SD *= .45) than non-responsible function (*M *= 2.68, *SD *= .45) teachers [*t*(_309_) = −2.20, *p *= .01]. It also exhibited a significant mean difference, such that more subjective experienced scored higher (*M *= 2.86, *SD *= .45) than less subjective experienced (*M *= 2.68, *SD *= .45) teachers [*t*_(210)_ = −8.66, *p *< .01].

**Discussion:**

Hypotheses regarding age and role tenure were in the expected direction, but non-significant. The MHL measure for teachers demonstrated sound measurement properties supporting usage across teaching levels.

## Introduction

The topic of mental health is generally playing an increasingly important role worldwide. In particular, children and adolescents are increasingly affected (1). Although exacerbated by the COVID-19 pandemic, the topic was also already gaining awareness at political and societal levels at-large (2).

The prevalence of psychological disorders in children and adolescents is estimated to be around 20% (3). The variety of psychological disorders in school-age ranges from oppositional defiance to ADHD to suicide ideation depending on the age and school level (4, 5). In general, schools are a very important environment for early identification of children at risk, and school teachers are often the first to recognize psychological distress and symptoms. However, the chance of early detection is underused, as school staff is rarely trained or confident to identify mental health symptoms or to promote mental health.

In Switzerland, schools are increasingly aware of their responsibility regarding health literacy of school children. Health learning objectives are included in the national teaching objectives, which states that “Health encompasses physical, mental and social well-being of humans” (6), and schools and teachers implement activities with the aim to strengthen health competencies. However, in a case report from Switzerland, Mattig reports that, although the majority of Swiss elementary schools actively promote students' mental health with various programs, they are often unaware of specific and concrete support programs with empirically proven effectiveness (7). Kunz and Luder point out that evidence-based activities are important, but that their adaptation to individual needs and settings is even more crucial (8). These programs typically focus on the children and or the school climate, rarely do they address mental health literacy of teachers or other school staff involved in care of children.

To date, only few studies have examined mental health literacy of teaching or school staff. However, some qualitative studies focused on knowledge and attitudes (9), but have not directly addressed the concept of mental health literacy. Correctly recognizing children's and adolescents' distress can overwhelm school staff (10). Similarly, a scoping review found that teachers and social workers were “uncertain” in dealing with affected students on the basis of concrete, daily-school routine (11). This was especially evident in connection with more latent symptoms (12).

Health literacy is defined as the competency to find and access, to understand and evaluate, and also to apply information regarding a health issue (13). Most health literacy studies identify the application to be the hardest and lowest scoring competency. Application is a complex competency, as it relies on the subjective confidence in understanding the issue, one's agency and acceptance of responsibility and role, as well as trust in the personal communication and action competencies*.* Mental health literacy essentially consists of the same competencies but in addition includes a more general understanding of mental health as a non-stigmatized condition that can be treated and overcome. In this context, subjective beliefs of people about mental health are often obstacles in recognizing, managing and preventing mental health disorders. Mental health literacy therefore also encompasses subjective beliefs and knowledge about mental health (14). However, we identified a lack of instruments to assess and compare mental health competencies. Mostly, mental health competencies are measured subjectively, where the risk of inherent bias has to be taken into consideration. For example, most instruments are either unvalidated or focus on knowledge to the neglect of practical application (15). Ahnert et al. developed an instrument to measure knowledge *and* action-oriented competencies, focusing on mental distress and depression (16). More concretely, their instrument was developed to evaluate a training program for teachers' improvement in recognizing mental distress and depression, as well appropriate reactions.

Our study among kindergarten, primary and secondary schools of Winterthur, Switzerland, provided the opportunity to examine an adapted version of the Ahnert et al. instrument, the Mental Health Action Competency (MHAC) instrument, its properties and sensitivity, as well as the application in different teaching levels. We hypothesized that the mental health competency score should positively associate with subjective experience with psychologically distressed or burdened children, as well as with confidence in managing such children and the situation. Also, we hypothesized a positive association with age, role tenure, and teachers' class-responsibility function within the schools.

## Materials and methods

### Study design and study population

The study targeted school teachers and other school professionals responsible for school children, either in a school classroom environment or in after-school care in compulsory schools of the city of Winterthur, Switzerland (inclusion criteria). In Switzerland schooling is compulsory from Kindergarten up to grade nine. A cross-sectional study was conducted from 26 February to 24 March 2020, and terminated prematurely due to the impact on schools by the Corona pandemic. The regional school authorities provided e-mail addresses of all school staff eligible for participation (*N* = 1,514) based on their mailing registry. An invitation with a survey link, an invitation letter signed by the school authorities and researchers, as well as study information was sent out and one reminder was sent two weeks after the initial mailing. From the 563 responding staff, 139 were excluded from the data set, due to non-eligibility, resulting in a data set of *N* = 425. Of these, a further *n* = 56 data were missing for the focal MHAC instrument, reducing our effective dataset to *N* = 369.

### Study questionnaire and measures

The online questionnaire was developed by the research team, consisting of pediatric, sociological, and public health experts in collaboration with school representatives (two directors of primary schools, a school social worker and an administrator responsible for local school development). When possible, validated instruments or items published in literature on mental health and mental health literacy were applied. Few questions were designed specifically for the study to address the local environment.

### Outcome measure

The MHAC scale applied, consists of 17 items covering statements on action competences, interaction competences and mental health knowledge. The scale is based on a scale implemented by Ahnert et al. in (16) focusing on depression in children and adolescents (16). We adapted the German questionnaire by Ahnert et al. to encompass a wider range of psychological disorders (see Appendix A). Responses are provided using a 4-point Likert-type scale ranging from 1 (not true at all) to 4 (fully agree), with total scores ranging from 17 to 68 points. Higher scores reflect higher competencies in detecting mental health issues in students.

#### Predictor measures

Socio-demographic and biographical participant data was collected to test hypotheses regarding discriminant validity of our outcomes measure and to assess its functioning across different school levels. School levels were kindergarten, primary school and secondary school. Further, this information was used for the external validation analyses regarding occupational characteristics.

### Statistical analyses

Descriptive analyses were performed for the whole sample. Data cleaning and classical analyses were conducted in software IBM SPSS v27. Specifically, maximum likelihood estimation was conducted in the factor analytics. IRT analyses were conducted in software IRTPRO v5.1 with specification using the graded response model. Three levels of the MHACS instrument are inspected for internal validation, specifically: (1) Scale dimensionality, (2) Subscale reliability, and (3) Item bias. Exploratory factor analyses were used to determine the dimensionality of the MHACS. Subscale reliabilities are computed with a bifactor indices calculator based in MS Excel (17). Item bias was examined with a two-step procedure, specifically: (1) Traditional statistical criteria for detection using chi-square values (*χ*^2^), and (2) Magnitude using calculations of McFadden's pseudo *R*^2^ statistic. We applied medicine's conventional criteria guideline to evaluate item bias magnitude as follows: <0.13 = negligible bias, 0.13–0.26 = moderate bias, and >0.26 = large bias (18). Lastly, group-mean differences were examined using independent *t*-tests for age, time-in-position, class responsibility, and mental health experience level.

The study protocol pertaining to the enclosed questionnaire administered to consenting adults was part of an internal-quality survey study, which is exempted from the Swiss Human Research Act (HFG). Data is available to interested researchers upon written request.

## Results

Univariate item-level descriptive statistics, frequency response patterns, and graphical inspection of scale-level normal Q-Q plots provided tentative evidence for inferring univariate-normal distributional assumptions. Specifically, all items' skewness (<2) and kurtosis (<7) values were within normality-range recommendations for large sample sizes (*n* > 300). Therefore, analyses proceeded with parametric tests. Summary sample descriptive characteristics are presented in Table 1 below. Analytic findings are organized by internal validation (scale, subscale, item) and external validation (age, role tenure, help function, and self-efficacy) sections below.

**Table 1:** Summary sample descriptive characteristics (*N* = 369).

Characteristic	*n* (%)
School level	
Kindergarten	76 (21)
Primary	210 (57)
Secondary	83 (23)
Sex	
Female	295 (80)
Male	72 (20)
Age category^a^	
<31 years	78 (21)
31–40 years	105 (29)
41–50 years	80 (22)
51–60 years	92 (25)
>60	13 (4)
Time in Position^a^	
Less: ≤10-year	227 (62)
More: >10-year	142 (39)
Class Responsibility	
Teacher w/o class responsibility	47 (15)
Teacher w/class responsibility	264 (85)
Subjective Experience w/Mental Health Issues^a^	
Less: none - little	79 (37)
More: experienced - very experienced	133 (63)

*N* = 369.

^a^ Indicates variables analysed via median-splits.

### Scale dimensionality

**Figure 1:**
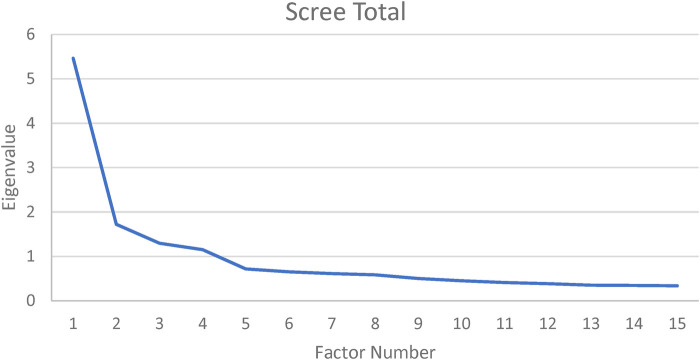
Scree plot of MHAC latent-factor structure. *N *= 369. Estimation = maximum likelihood.

Exploratory factor analysis (parallel analysis) was conducted to examine dimensionality of the MHAC instrument. First, a full factor-loading table is displayed in Appendix B, which illustrates appropriateness of factor-analytic methods as indicated by KMO value of .87 and Bartlett's sphericity test of *p *< .01. As shown in Figure 1 below, the first eigenvalues indicated the presence of a strong general factor. Specifically, the first and second eigenvalue ratio was 5.46/1.72 = 3.17, suggesting negligible multidimensionality (19). The high eigenvalue-ratio was replicated for each school level in our data, specifically: Kindergarten 5.17/1.61 = 3.21, Primary = 5.02/1.16 = 4.33, and Secondary 5.17/1.35 = 3.83.

A strong general factor was supported by a fairly high estimated common variance (ECV, .64), indicating that approximately 64% of all MHAC variance is explained by its general factor. Inspection of general factor loadings (*Λ*), however, revealed two items that were weak indicators: “I find it difficult to decide what help is appropriate for a student” *Λ* = .19; “Before I talk to the affected student myself, I would first inform the school psychologist” *Λ* = .04. Removal of the two items reduced the MHAC from 17 to 15 items, and ECV values were re-estimated, resulting in no reduction. Analyses proceeded with the 15-item MHAC instrument.

Further inspection of hierarchical-Omega (*ω*H: reliability) of the general factor complemented the eigenvalue ratios. Specifically, *ω*H = .88, supporting the interpretation of total scores as “essentially unidimensional” (20). Finally, comparing factor loadings across unidimensional and multidimensional models indicates the relative bias from fitting multidimensional data to a unidimensional model. The average-relative parameter bias value (.26) indicates that the impact of ignoring multidimensionality by using unidimensional MHAC scores may be substantive (21). In the next section, we further examine how substantive multidimensionality may be in our data by examining subscale reliabilities.

Although our ECV value was not as high as some traditional benchmarks (e.g., >.70), comprehensive inspection of our data satisfied claims regarding appropriate usage of MHAC as unidimensional. Specifically, Reise and colleagues state “when PUC values are lower than .80, general ECV values greater than .60 and OmegaH > .70 (of the general factor) suggest that the presence of some multidimensionality is not severe enough to disqualify the interpretation of the instrument as primarily unidimensional” (20; p. 22).

### Subscale reliability

Subscale reliability is estimated with model-based reliability coefficients. Specifically, omega coefficients (*ω*) are analogous to Cronbach's alpha (*α*) when multidimensionality's impact is unknown. We computed *ω* reliabilities and compared it to hierarchical-omegas (*ω*H), which represents “pure” subscale reliability, excluding the general factor. For clarity, subscale-specific reliabilities are labeled *ω*(Know) and *ω*(Act) to denote original subscale labels Knowledge and Action, respectively (16).

First, IRT estimates were used to compute subscale omegas as *ω*(Know) = .98 and *ω*(Act) = .99. Second, hierarchical-omegas were computed for each subscale as *ω*H(Know) = .46 and *ω*H(Act) = .11. This dramatic reduction in values already indicates that, after controlling for the general MHAC factor, little reliable variance remains for meaningful interpretation of subscores. Third, dividing the subscales’ *ω*H by their respective *ω* coefficients illustrates the percentage of reliable variance in subscales, excluding general MHAC. Calculating for Knowledge subscale (.46/.98 = .47) and Action subscale (.11/.99 = .11), it indicates that 47% and 11% of reliable variance in the subscales is independent of the general MHAC. This should be interpreted as insufficient reliability (<.70) for using MHAC subscores in educational research. For broader (more common) interpretability, Cronbach`s alpha for the overall 15-item MHAC instrument was estimated at *α* =.

### Item bias

IRT testing of measurement equivalence proceeds with assessment of differential item functioning (DIF), or, “item bias”, which Lord defined as parameter differences in an item's response function across nominal groups (e.g., Secondary, Primary, and Kindergarten school levels) (22). As mentioned, a two-step procedure was followed comprising: (1) statistical detection, and (2) magnitude assessment.

Results displayed in Table 2 below indicate two items in the primary-school sample detected for potential bias: Items 4 and 6. Review of item content indicated that item 4 relates to self-efficacy in approaching parents of suspected mental-health affected children, whereas item 6 relates more generally to self-efficacy on where to bring students suspected of mental-health affliction. McFadden's pseudo *R*^2^ was calculated to evaluate bias magnitude in order to determine the appropriateness of retaining the items for use in an educational context (23). As shown in Table 2's right column, all *R*^2^s were computed as below the .13 threshold, suggesting negligible bias and appropriateness of the items to remain in the MHAC for mental health competency assessment in primary school educational contexts. For Kindergarten level, no items were detected as exhibiting bias, suggesting that the MHAC instrument may be appropriate for administration in Kindergarten-level educational contexts.

**Table 2:** MHAC item-bias detection and magnitude evaluation.

Detection (Primary vs. Secondary)							Magnitude
Item #	Total *χ*^2^	*d.f.*	*p*	Slope *χ*^2^	*d.f.*	*p*	*McFadden's pseudo R^2^*
1	0.8	4	0.94	0	1	1.00	
2	4	4	0.40	3.3	1	0.07	
3	1.5	3	0.68	0.3	1	0.56	
4	20.4	4	0.00	6.7	1	0.01	.02
5	0.8	4	0.94	0.3	1	0.56	
6	20.9	4	0.00	7	1	0.01	.01
7	7	4	0.14	0.2	1	0.65	
8	5.7	4	0.22	0.6	1	0.44	
9	5.8	4	0.22	0.1	1	0.74	
10	9.3	4	0.05	0	1	0.85	
11	4.4	4	0.36	0.1	1	0.79	
12	2.9	4	0.57	1.4	1	0.24	
13	1.5	4	0.82	0.1	1	0.75	
14	1.4	4	0.85	0	1	0.84	
15	5.4	4	0.25	0.5	1	0.48	
Detection (Kindergarten vs. Secondary)							Magnitude
Item #	Total *χ*^2^	*d.f.*	*p*	Slope *χ*^2^	*d.f.*	*p*	*McFadden's pseudo R^2^*
1	2.2	4	0.69	0.8	1	0.36	
2	0.4	4	0.98	0.1	1	0.77	
3	3.3	3	0.35	0.6	1	0.45	
4	5.1	3	0.16	1.6	1	0.21	
5	4	4	0.41	0.8	1	0.38	
6	9.3	4	0.05	0	1	0.84	
7	6.6	4	0.16	0	1	0.96	
8	8	4	0.09	1.2	1	0.27	
9	5.3	4	0.26	0.5	1	0.50	
10	8.6	4	0.07	1.9	1	0.17	
11	6.1	4	0.19	0	1	0.88	
12	2	4	0.73	0.2	1	0.63	
13	2.7	4	0.61	0	1	0.93	
14	7.9	4	0.09	0.6	1	0.43	
15	3.1	4	0.55	0.1	1	0.78	

*N* = 369.

*χ*^2^ = 2 log likelihood. d.f, degrees of freedom. *McFadden's pseudo R^2^* computed as ratio of restricted model/free model using −2loglikelihood values.

## External validity

External validation hypotheses received mixed support. First, our hypothesis regarding higher MHAC scores with age was in the expected direction (*M*_young_ = 2.81, *M*_old_ = 2.86). Inspection of the *t*-test, however, indicated non-significance, *t*(367) = −.87, *p *= .19. Second, our hypothesis regarding higher MHAC scores with longer Role Tenure was also in the expected direction (*M*_ShortRole_ = 2.82, *M*_LongRole_ = 2.85). Inspection of the *t*-test, however, indicated non-significance, *t*(367) = −.60, *p *= .28.

**Figure 2:**
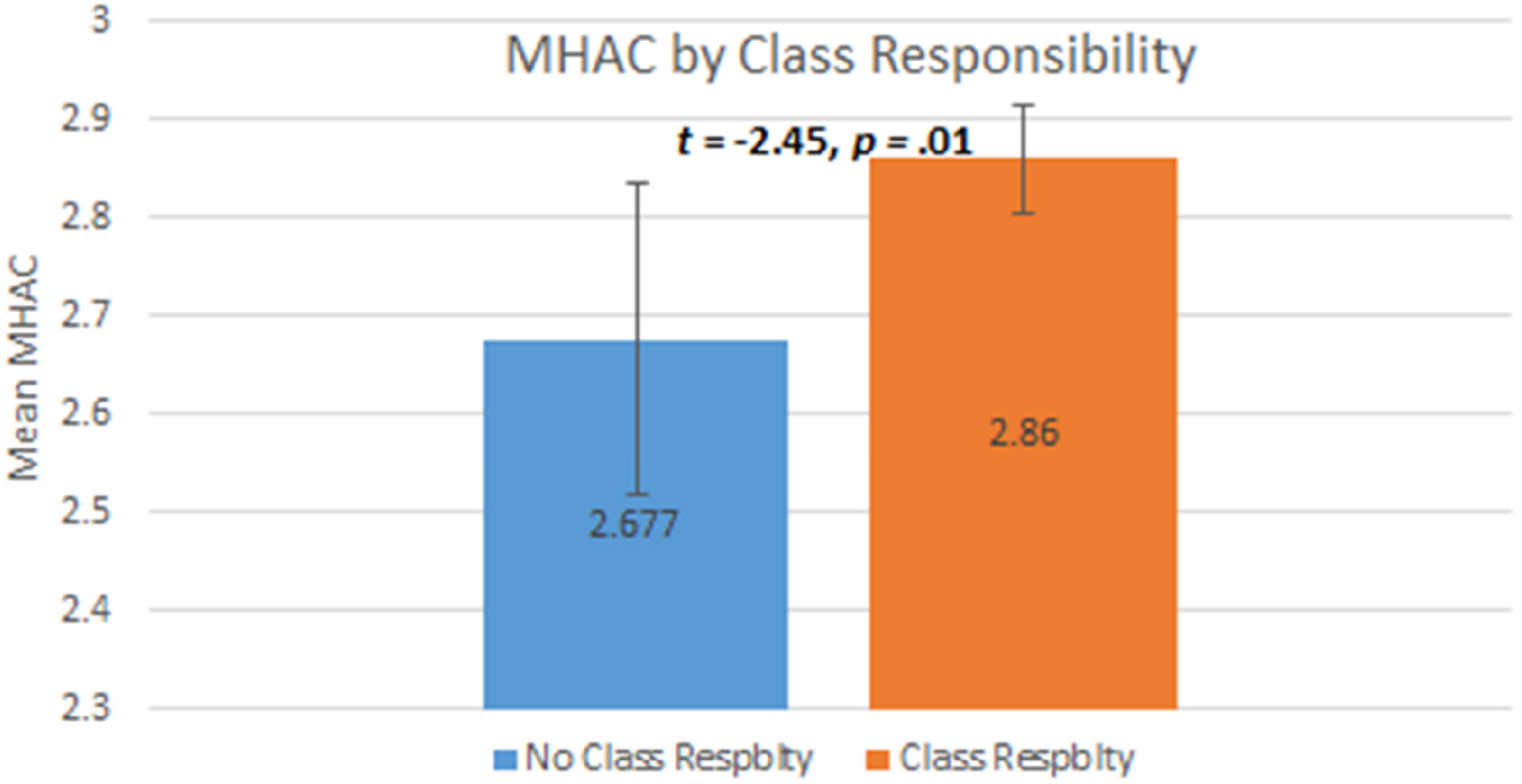
*N *= 369. 95% confidence intervals displayed. Respblty = Responsibility.

Third, our hypothesis regarding higher MHAC scores with class responsibility functions was supported. Specifically, participants in a Class-Responsibility Function reported significantly higher MHAC (*M* = 2.86) compared to those in a No-Class Responsibility Function (*M* = 2.68); *t*(309) = −2.20, *p *= .01. Bar-chart illustration of this supported hypothesis is depicted in Figure 2 below.

**Figure 3:**
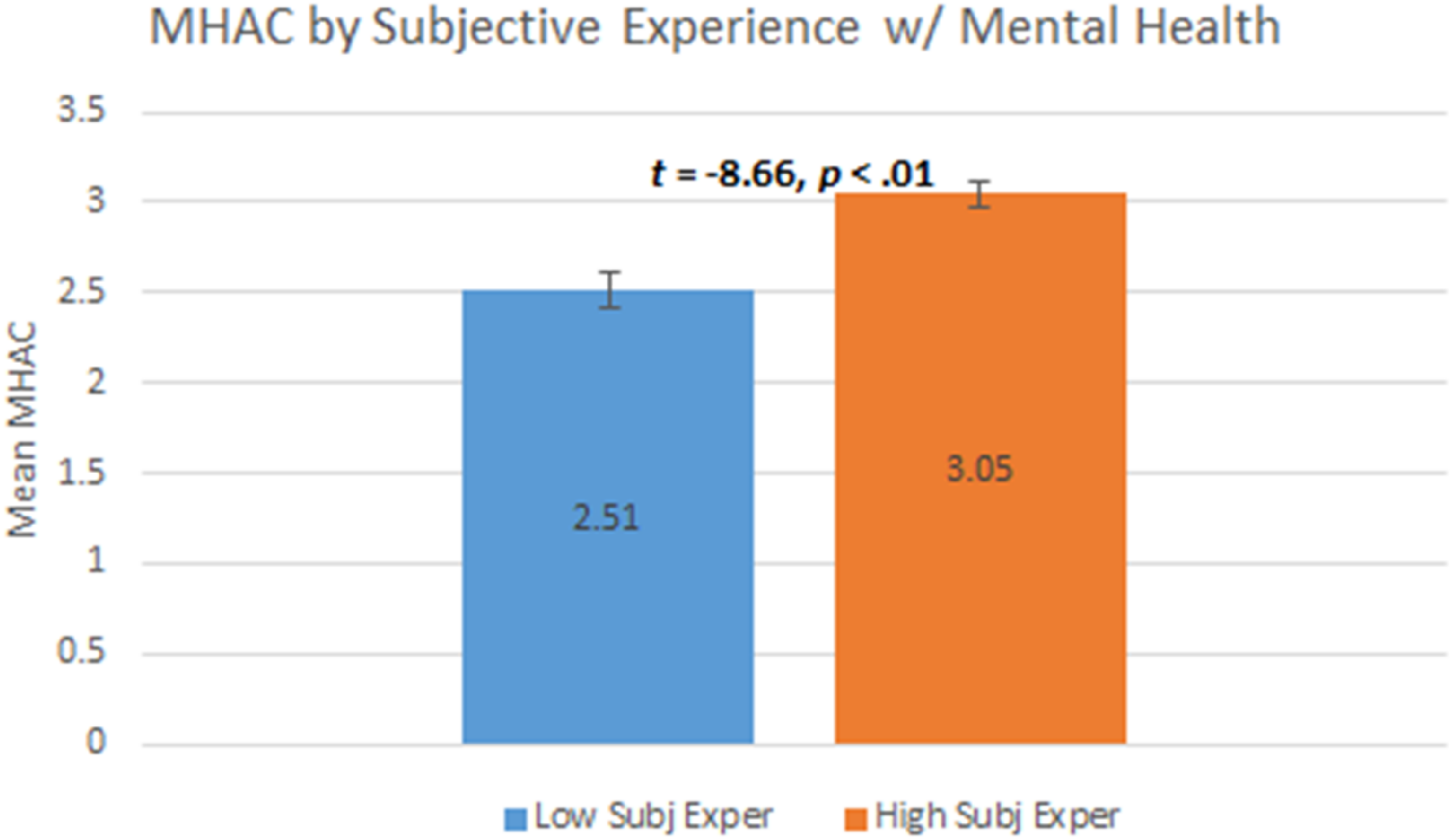
*N *= 311. 95% confidence intervals displayed. Subj Exper = subjective experience.

Finally, our hypothesis regarding higher MHAC scores with higher subjective experience with mental health issues was also supported. Specifically, participants with higher Self-efficacy reported significantly higher MHAC (*M* = 3.05) compared to those with lower Self-efficacy (*M* = 2.51); *t*(210) = −8.66, *p *< .01. Bar-chart illustration of this supported hypothesis is depicted in Figure 3 below.

## Discussion

This study sought to bring evidence from mental health “calls-to-action” to bear on a developed competence self-report (16). Specifically, we aimed to extend evidence from a pre-post study of secondary teachers (responsiveness evidence) to a cross-sectional study across multiple school levels (sensitivity evidence). Several hypotheses were formulated that spanned internal-psychometric validation and external-correlates validation (24). Test results of hypotheses are summarized below.

First, regarding dimensionality, our results indicated that the MHAC exhibited a strong general factor. This was further supported by a high omega-reliability for the MHAC total score, which supports the instrument's “essential unidimensionality” (20). Therefore, MHAC total scores are supported as unidimensional construct indicators in future research reports. Two items that were reverse-coded and ambiguous were identified as weak indicators of the general factor and are recommended for removal. In fact, Ahnert et al. also identified the same items as weak with little change (low responsivity) over time (16).

Second, regarding subscale-reliability, our current findings failed to support the future reporting of MHAC subscales. Specifically, calculation of subscale-hierarchical omegas indicated insufficient reliability for interpretation as individual-difference indicators. It may be noted that our subscales were based on original constructors’ knowledge- and action-based conceptualizations (16). Future researchers may consider adding items to specific subscales to attain acceptable empirical reliabilities for specific-subscale interpretation.

Third, regarding item-bias, IRT DIF analyses indicated two items exhibiting statistical significance in the primary-school sample. Further inspection and computation of substantiveness indicated that the DIF was negligible (18). Furthermore, we found no indicator of DIF in our kindergarten sample. While the original instrument was developed for teachers of adolescents, our findings supports the extension of MHAC applications to primary school samples and tentative support for the use of the MHAC among personnel also at kindergarten-level schools. We also found acceptable psychometrics to verify MHAC's use at secondary school levels.

Finally, regarding external validation via inspection of postulated correlates, our hypotheses received mixed-support. Our two hypotheses regarding Age and RoleTenure were in the expected direction, but failed to reach significance. In contrast, our two hypotheses regarding Class Responsibility and Subjective Experience were supported. Aside from the known lack of power involved in median-splits hypothesis testing, failure to find support for our more temporal-demographic variables may be partly explained by a period/cohort effect (25). One might assume that teachers acquire mental health competencies throughout their tenure. It is also important to note that interest and motivation of teachers is a strong predictor of engagement with students in general (26). With regard to our results, there possibly is too little further education provided for teachers on the topic or the recent increased awareness of mental health issues in the general population may suppress the effects for higher MHAC associated with Age and RoleTenure. Our cross-sectional design further exacerbates the separability issue of age-period-cohort effects (27).

### Limitations and future directions

It should be noted that the two weak items removed during our dimensionality inspection may be retained for specific purposes in future research. For example, one of the items is the only reverse-coded item and, therefore, may be retained as a quasi-response quality indicator (to screen for inattentive responding). The second – ambiguous – item may be inapplicable in general, but it also may serve as a structural indicator of resource constraints at particular schools. For example, if psychologists are not normally employed at kindergarten-level schools, then exclusion of the item is sensible. However, if samples include school-levels typified by employed school psychologists, then retention of the item may serve as a value indicator as to teacherś knowledge or awareness regarding this resource.

The presented findings indicate that the instrument may be used to screen mental health literacy action competencies in teachers and pedagogical professionals at different school levels. It may help to identify in which area teachers will profit most from training and allow for focused programs and interventions. Further research on the instrument's external validity is merited, however. Given the high importance of the school setting to strengthen children's mental health and for early identification of at-risk children, assessments of mental health competencies must be followed by concrete training and information targeting teacher's resourceful responsiveness.

## Data Availability

The raw data supporting the conclusions of this article will be made available by the authors, without undue reservation.

## Appendix A

Original and Adapted Items of the Mental Health Action Competencies (MHAC) Instrument.

**Table:**

Item #	Item Content
1	I have the confidence to take action if I suspect that a student is at increased risk of mental stress, disorders or illnesses.
2	I have the confidence to approach the student personally.
3	I have the confidence to approach parents about the signs.
4	I know when it is appropriate to involve a specialist when dealing with a student with a mental stress, disorder or illness.
5	I know where to refer students with increased risk for mental stress, disorders or illnesses if necessary.
6	I know where to turn when I need support in dealing with students at increased risk for mental stress, disorders or illnesses.
7	*“I find it difficult to decide what help is appropriate for a student”*
8π	I have the confidence to convince what I believe to be a mentally distressed or depressed student that he/she should seek help.
9	*Before talking to the affected/troubled student myself, I would first inform the school psychologist.*
10π	I have the confidence to be able to distinguish whether a student is sad or depressed.
11	I am confident in my ability to distinguish impulsive or nervous behavior from attention deficit hyperactivity disorder (ADHD).
12	I trust myself to be able to distinguish anxious behavior from an anxiety disorder.
13***	I am familiar with the main warning signs that indicate suicidality in a student in the school context.
14	I have the confidence to recognize when a student has an eating disorder.
15*	I am confident in recognizing when a student has a problem with addictive substances such as cannabis, nicotine, alcohol.
16*	I have the confidence to recognize when a student has a problem with their online media use, online gambling or illegal gambling?
17*	I have the confidence to take action if I suspect that a student is at increased risk of mental stress, disorders or illnesses.

* Indicates new items added to original instrument from Ahnert et al. (16), π item was excluded based on factor loadings.

## Appendix B

Item-Factor Pattern Loading Table of 17 MHAC Items.

**Table:**

Factor matrix^a^	Factor1
Handlungskompetenz 1	.623
Handlungskompetenz 2	.549
Handlungskompetenz 3	.391
Handlungskompetenz 4	.452
Handlungskompetenz 5	.629
Handlungskompetenz 6	.511
Handlungskompetenz 7	.481
Handlungskompetenz 8	−.153
Handlungskompetenz 9	.579
Handlungskompetenz 10	.131
Handlungskompetenz 11	.596
Handlungskompetenz 12	.582
Handlungskompetenz 13	.643
Handlungskompetenz 14	.597
Handlungskompetenz 15	.612
Handlungskompetenz 16	.620
Handlungskompetenz 17	.547

Extraction Method: Maximum Likelihood.

^a^ 1 factors extracted. 4 iterations required.

**Table:**

KMO and Bartlett's Test		
Kaiser–Meyer–Olkin Measure of Sampling Adequacy.		.868
Bartlett's Test of Sphericity	Approx. *χ*^2^	2,084.734
	df	136
	Sig.	<.001
